# Physiological Implications of Pancreatic Amyloid Polypeptide Aggregation and Its Inhibition by Melatonin

**DOI:** 10.3390/ijms27062910

**Published:** 2026-03-23

**Authors:** Yeong-Min Yoo, Seong Soo Joo

**Affiliations:** 1 Institute of Environmental Research, Kangwon National University, Chuncheon 24341, Republic of Korea; 2 Department of Marine Bioscience, College of Life Science, Kangwon National University, Gangneung 25457, Republic of Korea

**Keywords:** human islet amyloid polypeptide (hIAPP), type 2 diabetes (T2D), Alzheimer’s disease (AD), type 3 diabetes (T3D), melatonin, protein aggregation, beta-sheet formation, amyloid inhibition

## Abstract

Type 2 Diabetes (T2D) is characterized by the toxic aggregation of human islet amyloid polypeptide (hIAPP or amylin) within pancreatic β-cells. IAPP is also a neuropancreatic hormone that plays a significant role in Alzheimer’s disease (AD) by co-depositing with amyloid-beta (Aβ) and Tau, supporting the Type 3 Diabetes (T3D) hypothesis. Soluble IAPP accelerates Aβ aggregation through cross-seeding and causes neurotoxicity by impairing the blood–brain barrier and activating neuroinflammation. Melatonin inhibits these processes by disrupting hydrophobic interactions in both hIAPP and Aβ, preventing the formation of toxic β-sheet structures. Furthermore, melatonin promotes amyloid clearance via the glymphatic and lymphatic systems, protects neurons from oxidative damage, and reduces Tau hyperphosphorylation. This suggests that melatonin serves as a promising multitarget therapeutic agent for both metabolic and neurodegenerative disorders by modulating structural protein transformations.

## 1. Introduction

Protein misfolding and subsequent aggregation are fundamental pathological processes underlying a diverse group of debilitating conditions known as amyloidopathies or protein-misfolding diseases. These conditions are typified by the aberrant self-assembly of ordinarily soluble proteins or peptides into insoluble, highly ordered fibrillar structures that are clinically significant and are termed amyloid fibrils [1]. A distinguishing feature of amyloid fibrils, irrespective of their constituent protein, is the prevalence of a predominant β-sheet secondary structure [1,2]. This structural motif facilitates extensive hydrogen bonding along the fibril length, generating a distinct cross-β x-ray diffraction pattern [3]. This invariant structure facilitates the specific binding of histological stains, such as thioflavin S and Congo Red, enabling their visualization by fluorescence microscopy and polarized light, respectively [4,5].

Recent research has indicated that the cytotoxic species responsible for cell death in amyloid diseases often arise during the early stages of aggregation, when small soluble oligomers and protofibrils begin to form. These early aggregates, rather than mature fibrils, are increasingly recognized as the primary toxic entities [5,6]. Furthermore, these small cytotoxic aggregates from a variety of amyloidogenic precursor proteins adopt a common structural conformation, regardless of their specific amino acid sequences [5,6]. This observation, that all amyloid fibrils share a common cross-β structure, and more importantly, that the toxic early aggregates from different amyloidogenic proteins adopt a common structural conformation, suggests that the underlying mechanisms of cellular toxicity are conserved across various amyloidopathies rather than being unique to each specific protein [1,2,3]. This commonality implies a shared vulnerability across these diverse conditions, presenting a significant opportunity for developing universal or “pan-amyloid” therapeutic strategies [7]. Instead of designing drugs specific to each amyloidogenic protein, interventions can target the shared toxic structural motifs, potentially leading to more broadly effective and efficient treatments for a range of protein misfolding disorders [8,9].

Numerous amyloid diseases with over 30 distinct conditions are linked to abnormal protein folding and conversion to an insoluble state [10]. These include major global health burdens, such as Alzheimer’s disease (AD), type 2 diabetes (T2D), Parkinson’s disease, and prion diseases, among others [5]. The pathological consequences of this process range from localized tissue damage, as evidenced in the case of pancreatic islets in T2D, to widespread systemic involvement affecting multiple organs and specific neurological degeneration in conditions such as AD.

This review focuses on human amylin, or islet amyloid polypeptide (IAPP), a 37-amino acid hormone physiologically co-secreted with insulin by pancreatic β-cells [11,12]. Although essential for glucose homeostasis, IAPP is highly amyloidogenic in humans, with a strong propensity to misfold and aggregate [11]. This aggregation leads to intracellular aggregate accumulation and extracellular amyloid structures that are closely linked to β-cell dysfunction and death in T2D [11]. Beyond its established role in pancreatic pathology, recent evidence—particularly within the last five years—has highlighted a significant and increasingly recognized pathological role of IAPP in cognitive function and neurodegenerative diseases, most notably in AD [11]. This dual involvement positions IAPP in the nexus of metabolic and neurodegenerative disorders. The consistent emphasis across multiple recent papers on IAPP’s pathological involvement in both pancreatic β-cell death in T2D and its direct contribution to cognitive impairment and AD pathology—including co-deposition with amyloid-beta (Aβ) and Tau—suggests that IAPP acts as a molecular bridge, connecting peripheral metabolic dysfunction (e.g., diabetes) directly to central nervous system degeneration. This finding supports the proposed designation of AD as type 3 diabetes (T3D) [11,13,14]. This connection implies that therapeutic strategies targeting IAPP aggregation or its downstream effects could offer synergistic benefits for both diabetes management and AD prevention or treatment. This underscores the importance of a holistic approach to patient care, recognizing that metabolic health profoundly impacts brain health and vice versa, and opens avenues for repurposing existing drugs.

Melatonin, a neurohormone primarily produced by the pineal gland, regulates circadian rhythm and sleep [15]. Beyond its chronobiotic functions, melatonin possesses potent antioxidant and anti-inflammatory properties [16]. A substantial amount of research, notably from 2019 to 2025, has demonstrated the remarkable capacity of melatonin to modulate protein aggregation, including that of amyloidogenic peptides such as IAPP and Aβ. The present study investigates the mechanisms by which melatonin exerts anti-amyloidogenic effects and evaluates its potential as a therapeutic agent for amyloid-related diseases. The primary objective of this study was to synthesize and critically analyze academic papers published in the past few years (2019–2025), focusing on the physiological and pathophysiological implications of IAPP aggregation and the multifaceted role of melatonin in its inhibition. The scope encompasses the molecular, cellular, and systemic levels of understanding, drawing on insights from in vitro and in vivo (animal and human) studies.

## 2. Physiological and Pathophysiological Significance of IAPP Aggregation

### 2.1. IAPP’s Normal Physiological Functions

IAPP, or amylin, is a 37-amino acid hormone that is co-secreted with insulin from pancreatic β-cells in a tightly controlled manner, typically in response to glucose stimulation [11,17]. This co-secretion is crucial for the maintenance of postprandial glucose homeostasis. Physiologically, IAPP contributes to glucose regulation through several key actions. It inhibits insulin and glucagon secretion, particularly nutrient-stimulated glucagon secretion, thereby reducing hepatic glucose output [11,17,18]. Additionally, IAPP plays a significant role in controlling adiposity and satiation by slowing gastric emptying, which in turn reduces the rate of glucose appearance in the blood and overall food intake, contributing to body weight regulation [11,17,19]. In the circulation, IAPP and insulin act synergistically to stimulate the uptake of blood glucose into muscle and adipose tissues, thereby stabilizing blood glucose levels in the postprandial period [11,17,20].

In addition to its direct role in glucose metabolism, IAPP exerts broader systemic effects. It is involved in blood pressure regulation and has a notable influence on the renin–angiotensin system [11,21]. Additionally, IAPP contributes to vasodilation and directly stimulates blood glucose uptake [11,22]. These systemic actions underscore multifaceted physiological importance of IAPPs.

### 2.2. IAPP Aggregation in T2D: A Pancreatic Perspective

Islet amyloid deposition is a hallmark pathological feature observed in the pancreatic islets of approximately 90% of individuals with T2D [5,23,24,25]. These deposits are primarily composed of misfolded IAPP [5,26,27]. Autopsy studies in humans have consistently demonstrated a strong association between the presence of islet amyloid and a significant loss of β-cell mass [5]. This loss of insulin-producing β-cells is a critical factor in the progression from insulin resistance to overt T2D [28]. The formation of islet amyloid directly contributes to β-cell dysfunction and death in T2D. Moreover, it is also implicated in the failure of islet transplants, which are therapeutic options for severe diabetes [11].

The aggregation process of IAPP begins with its misfolding from a native soluble form, progressing through the formation of highly toxic oligomers and protofibrils, which then mature into larger amyloid fibers [11,29,30]. Experimental studies have shown that smaller IAPP oligomers exhibit higher cytotoxicity than larger aggregates or mature fibrils [31]. This toxicity is mediated by a complex interplay between molecular and cellular mechanisms.

■ Membrane Disruption and Ion Channel Formation: IAPP aggregation directly damages β-cells through membrane disruption [31,32,33]. At cytotoxic concentrations, IAPP forms voltage-dependent, relatively nonselective, ion-permeable channels in planar lipid membranes [34]. Channel formation depends on lipid membrane composition, ionic strength, and membrane potential, strongly suggesting that membrane permeabilization and ion dysregulation may be the primary mechanisms of IAPP-induced cytotoxicity [15].
■ Generation of Reactive Oxygen Species (ROS) and Oxidative Stress: IAPP aggregation generates oxidative stress, leading to significant oxidative stress within β-cells [32,35]. Oxidative damage contributes to cellular dysfunction and death.
■ Endoplasmic Reticulum (ER) Stress and Unfolded Protein Response: ER stress is a proposed mechanism contributing to IAPP-induced β-cell pathology [33,35]. IAPP aggregation enhances ER stress, which, in turn, generates additional autoantigens and intensifies the autoimmune response often observed in diabetes [31,33].
■ Activation of Inflammasome and Inflammatory Cascades: The activation of the inflammasome, a multiprotein complex that triggers inflammatory responses, has been proposed to play a role in IAPP-induced toxicity [32,35]. The accumulation of IAPP aggregates creates a feedback loop that promotes a chronic inflammatory environment within the islets, thereby perpetuating β-cell damage and destruction [31,32]. Inflammation can escalate into an inflammasome cascade, leading to organ damage.
■ Defects in Autophagy and Proteasome Dysfunction: Defects in cellular waste disposal systems, specifically autophagy, have been implicated in IAPP-induced β-cell death [33,35,36,37]. Furthermore, proteasome dysfunction linked to chronic inflammation and metabolic stress is exacerbated by IAPP aggregation, which impairs the degradation of misfolded IAPP and promotes the accumulation of toxic intermediates [31,36].
■ Receptor-Mediated Mechanisms: Receptor-mediated pathways have also been proposed to contribute to IAPP-induced β-cell toxicity [35,38].
■ Apoptosis: IAPP oligomers are a direct cause of β-cell apoptosis [33,39]. Metabolic stressors such as glucotoxicity, lipotoxicity, and chronic inflammation further exacerbate the burden on β-cells, accelerating this process [31].

The proposed mechanisms of IAPP-induced β-cell death, membrane disruption, ROS, ER stress, inflammasome activation, autophagy/proteasome dysfunction, and apoptosis indicate that β-cell demise is not due to a single pathway but rather to a complex, interconnected pathological network. For example, membrane disruption [15,31,32,33] can lead to ion imbalance, triggering ER stress, which, in turn, generates ROS and impairs proteostasis [31]. This creates a vicious cycle in which each mechanism exacerbates the others, thereby amplifying β-cell damage [35]. This intricate interplay suggests that therapeutic strategies aimed at protecting β-cells from IAPP toxicity may need to adopt a multitarget approach, rather than focusing on a single mechanism.

The critical role of unprocessed IAPP forms (proIAPP) in initiating aggregation has been increasingly recognized. IAPP is initially synthesized as an 89-residue precursor, pre-proIAPP, which is then processed to proIAPP after signal peptide cleavage in the ER [11], which is subsequently matured in the late Golgi complex by protein convertases (PCs) 1/3 and 2, which cleave flanking peptides to yield active, mature IAPP [11,40]. Under pathological conditions, particularly in prediabetic and diabetic phenotypes, there is an increased demand for insulin production, accompanied by elevated IAPP levels [11,12,41,42]. This overload and impairment of the β-cell processing machinery, especially enzymes that convert precursor molecules, leads to the accumulation of unprocessed IAPP forms. Recent studies have strongly suggested that the unprocessed IAPP forms, particularly proIAPP, serve as seeding agents for amyloid formation [11,12,41,42]. The accumulation of immature IAPP species and the formation of toxic intracellular oligomers have been directly associated with β-cell dyshomeostasis and apoptosis [11]. Elevated proIAPP levels and amyloid deposition have been observed in β-cells lacking PC1/3 and PC2, and proIAPP has been found in intracellular fibrils [11,12,41,42]. Impaired processing of proIAPP at its N-terminal cleavage site is considered a key factor in the initiation of amyloid formation, as this processing step occurs later in the secretory pathway and is more susceptible to impairment under high secretory demand [43,44,45]. The detailed understanding that unprocessed IAPP forms, particularly proIAPP, act as critical seeding agents and highly toxic intracellular oligomers, represents a significant shift in understanding the initiation of IAPP amyloidogenesis [11,12,41,42]. This implies that the problem begins before mature IAPP aggregates. This understanding points to a more upstream therapeutic strategy: preventing the accumulation of proIAPP or promoting its proper processing. This could involve the development of modulators of protein convertases (PC1/3 and PC2) or enhancement of cellular mechanisms for clearing these precursor forms. Targeting proIAPP could halt amyloid formation at its earliest and most vulnerable stages, offering a novel avenue for the prevention and treatment of T2D.

Crucial animal studies, including those using transgenic models of islet amyloid formation, have demonstrated that IAPP amyloid deposits form within the pancreatic islets before the onset of fasting hyperglycemia [5]. This provides compelling evidence that islet amyloid is not merely a consequence of diabetes but rather plays a causative role in the pathogenesis of T2D. The extent of IAPP amyloid deposition has been consistently associated with both the loss of β-cell mass and impairment of insulin secretion and glucose metabolism [5].

β-cell dysfunction appears to be an important prerequisite for islet amyloid formation [5]. Conditions that increase the secretory demand on β-cells, such as obesity and/or insulin resistance, further exacerbate IAPP amyloid deposition [5]. In the state of insulin resistance, the inability of the body to use insulin effectively leads to a compensatory increase in insulin production. Because proinsulin and proIAPP are co-secreted, this results in a concomitant increase in proIAPP production [41,46]. The overload of the processing machinery contributes to the accumulation of amyloidogenic proIAPP intermediates [11,12,41,42]. The data reveal a detrimental feedback loop: metabolic stressors, such as obesity and insulin resistance [5], increase secretory demand on β-cells, leading to elevated production of IAPP and its unprocessed forms [11,12,41,42]. This overload impairs processing, causing accumulation of toxic proIAPP and IAPP aggregates, which then directly damage β-cells, further compromising insulin secretion and worsening hyperglycemia [5]. This creates a self-perpetuating cycle that accelerates the disease progression. This underscores the critical importance of early interventions targeting metabolic health, including obesity management and improvement of insulin sensitivity. Such strategies are not only vital for glucose control but are also foundational for preventing the initiation and progression of IAPP amyloidosis, thereby preserving β-cell function and potentially averting the onset of T2D.

### 2.3. IAPP’s Pathological Role in Neurodegenerative Diseases: The “T3D” Hypothesis

In addition to its pancreatic effects, mounting evidence suggests that IAPP plays a substantial pathological role in cognitive function [11,12,41,42]. IAPP has been shown to interact and co-deposit with Aβ, the primary component of amyloid plaques in AD, and possibly with Tau protein within the brains of AD patients [11,47,48,49]. This co-pathology contributes significantly to the development of diabetes-associated dementia. This strong association has led to a compelling hypothesis that AD may, in part, result from metabolic dysfunction in the brain, supporting its proposed designation as T3D [11,47,48,49,50,51]. The co-deposition of IAPP and Aβ42 has been proven to contribute to AD onset and progression [11,12,41,42,52].

Mechanisms of IAPP-mediated neurotoxicity include:

■ Impairment of the Blood–Brain Barrier (BBB): IAPP impairs the integrity and function of the BBB [11,12,41,42]. Membrane-permeable IAPP oligomers may compromise the BBB, facilitating their diffusion into the brain parenchyma, thereby increasing their direct neurotoxic effects [53,54]. In brain microvascular pericytes of patients with AD and T2D, IAPP forms intracellular toxic inclusions [53,54].
■ Direct Interaction and Cross-Seeding with Aβ: Molecular Insights into Accelerated Aggregation: Amyloid aggregation of Aβ and IAPP are distinct but pathologically linked hallmarks of AD and T2D, respectively [47,50,52,53,54,55]. Recent studies have provided crucial molecular insights into their crosstalk: soluble IAPP can significantly accelerate Aβ aggregation [47,50,52,53,54]. It is evident that the acceleration is more pronounced in soluble IAPP than in preformed IAPP amyloids, which are poor seeds for Aβ aggregation [47,50,52,53,54,55]. The proposed mechanism involves a binding-induced conformational change within the amyloidogenic core of Aβ42, which subsequently reduces the aggregation free-energy barrier for Aβ, thereby accelerating its aggregation [47,50,52,53,54,55]. Specifically, the formation of an Aβ–IAPP heterodimer induces helix unfolding of Aβ16−22, resulting in accelerated coaggregation in comparison with Aβ42 alone [47,50,52,53,54,55]. While both Aβ and IAPP can form amyloid fibrils with similar cross-β structures, IAPP generally aggregates faster in vitro [47,50,52,53,54,55]. The discovery that soluble IAPP, as opposed to aggregated IAPP, is a significant accelerator of Aβ aggregation represents a critical mechanistic revelation [47,50,52,53,54,55]. This finding indicates that even minor elevations or dysregulation of soluble IAPP, which can occur in the early stages of T2D, may directly prime or exacerbate Aβ pathology in the brain. The underlying molecular mechanism is intricate and involves binding-induced conformational changes and reduced aggregation energy barriers. These characteristics provide specific targets for intervention [47,50,52,53,54,55]. This finding reinforces the “T3D” hypothesis by establishing a direct molecular pathway linking peripheral metabolic dysfunction (hyperamylinemia) to central neurodegeneration (Aβ accumulation). This suggests that regulating IAPP levels and preventing its initial misfolding or interactions with Aβ could be a potent strategy for preventing AD, particularly in individuals with T2D. This finding indicates that therapeutic approaches should prioritize the soluble, early-stage interactions between IAPP and Aβ.
■ Activation of Glial Cells and Neuroinflammation: Both individual IAPP aggregates and IAPP–Aβ co-aggregates are prone to activating glial cells (microglia and astrocytes) within the brain [53,56]. In response, these activated glial cells produce and release inflammatory mediators, such as cytokines, creating a chronic pathological environment that is highly detrimental to neurons [53,56].
■ Modulation of Neuronal Receptors (e.g., AMY3) and Synaptic Dysfunction: IAPP is capable of directly interacting with neurons, exerting its effects on specific neuronal receptors, including AMY3 [53]. Activation of AMY3 neuronal receptors by IAPP and Aβ elevates cytosolic cAMP, which, in turn, activates multiple signaling pathways (e.g., PKA, MAPK, AKT, and cFos). These pathways have been implicated in neuroinflammation, Aβ pathology, and neuronal cell death [53]. They can disrupt Ca^2+^ influx and ER homeostasis, thereby contributing to neuronal apoptosis [53,57]. At elevated concentrations, IAPP modulates signaling cascades that disrupt long-term potentiation. This, in turn, can result in synaptic failure [53,58].
■ Contribution to Diabetes-Associated Dementia: The cumulative effects of IAPP’s pathologies in the brain, including BBB impairment, direct neurotoxicity, and exacerbation of Aβ pathology, significantly contribute to the development and progression of diabetes-associated dementia [11,12,41,42].

Table 1 summarizes all the information about molecular and cellular mechanisms of IAPP-induced pathogenesis.

## 3. Melatonin: A Multifaceted Modulator of Amyloid Pathology

### 3.1. Overview of Melatonin’s Physiological Roles and Anti-Oxidative Properties

Melatonin, a hormone primarily synthesized by the pineal gland, is the key endogenous signal for darkness and plays a central role in regulating circadian rhythms and the sleep–wake cycle [15,59,60,61,62]. Importantly, clearance of amyloid aggregates from the brain is most effective during slow-wave sleep, a period characterized by high levels of adenosine triphosphate (ATP) and melatonin [63,64]. Evidence suggests an explicit link between melatonin, slow-wave sleep, and effective amyloid clearance, thus elevating the role of melatonin beyond that of a simple anti-aggregant. The hypothesis is that melatonin functions as a systemic orchestrator of proteostasis, primarily by optimizing the natural waste clearance mechanisms of the brain, such as the glymphatic system, which is most active during sleep. The observation that diminished ATP and melatonin levels during inadequate sleep result in incomplete clearance underscores this pivotal feedback loop [63,64]. This understanding emphasizes that sleep disturbances, a prevalent comorbidity in aging and neurodegenerative diseases, are not just symptoms but potentially direct drivers of amyloid pathology by impairing clearance of amyloid aggregates. Therefore, therapeutic strategies for amyloidopathies should consider not only direct molecular interventions but also the restoration of healthy sleep patterns and circadian rhythms. Melatonin may serve as a key pharmacological or lifestyle intervention to support endogenous clearance processes.

Beyond its chronobiotic functions, melatonin is recognized as a potent antioxidant and anti-inflammatory agent [16]. It directly protects neuronal cells from Aβ-mediated oxidative damage and reduces abnormal protein nitration, a marker of oxidative stress [65,66,67]. Moreover, melatonin may attenuate systemic inflammation and oxidative stress, which are the hallmarks of numerous pathological conditions [16,68].

### 3.2. Mechanisms of Melatonin’s Anti-Amyloidogenic Action

#### 3.2.1. Direct Inhibition of IAPP Aggregation

Recent molecular dynamics simulations, particularly replica-exchange molecular dynamics, have provided atomic-level insights into the direct inhibition of hIAPP aggregation by melatonin [69,70]. These simulations demonstrate that melatonin molecules significantly prevent the formation of β-sheets and backbone hydrogen bonds within the amyloidogenic hIAPP20–29 octamer [70,71]. This is of pivotal significance because the formation of β-sheets is a prerequisite for amyloid fibril assembly [5]. Melatonin interacts with hIAPP, resulting in remodeling of hIAPP oligomers into less compact conformations characterized by increased disorder [70,71]. This structural alteration is hypothesized to render the oligomers less prone to further aggregation and potentially less toxic.

Detailed interaction analyses reveal that melatonin binding to hIAPP is primarily driven by hydrogen bonding with the peptide backbone. The interaction is further strengthened by specific aromatic stacking and CH–π interactions with the side chains of amino acids within the peptide [70,71]. Melatonin preferentially binds to the critical amyloidogenic region of hIAPP, specifically hIAPP20–29 [70,71]. This strong and specific interaction disrupts the self-association of hIAPP20–29, which is hypothesized to inhibit overall amyloid aggregation and the associated cytotoxicity [70,71]. Detailed molecular simulations reveal specific interactions between melatonin and the hIAPP20–29 amyloidogenic region, its ability to prevent β-sheet formation, and the remodeling of oligomers into less compact structures. The finding that it exerts a negligible influence on preformed fibrils is of critical significance [70,71]. This finding suggests that melatonin does not function as a “fibril breaker” but rather as an “aggregation blocker,” exhibiting optimal efficacy at the earliest stages of the amyloid cascade. The ability to precisely target early toxic intermediates is significant. This mechanistic understanding provides critical guidance for the optimal timing of melatonin intervention in amyloidopathies, and is most beneficial as a preventative agent or in the very early stages of the disease, before extensive fibril deposition occurs. This also indicates that for advanced amyloidopathies, the efficacy of melatonin may be optimized by combining it with agents that disaggregate mature fibrils, thereby creating a multifaceted therapeutic strategy.

#### 3.2.2. Modulation of Aβ Aggregation and Clearance

Melatonin inhibits the formation of amyloid fibrils in vitro [65]. More importantly, in vivo studies using transgenic mouse models of Alzheimer’s amyloidosis (e.g., Tg2576 mice) have demonstrated that melatonin administration partially inhibits the time-dependent increase in Aβ in the brain [65]. A reduction in hippocampal Aβ levels has also been demonstrated, with concomitant improvements in short-term memory observed in streptozotocin-induced models of sporadic AD [68]. Melatonin actively protects neuronal cells from the oxidative damage mediated by Aβ [65,72,73,74]. This finding is consistent with its established role as a radical scavenger [15]. Beyond Aβ, melatonin may also reduce Tau protein hyperphosphorylation [68,75,76], thereby addressing another key pathological hallmark of AD. The effects of melatonin on AD pathology extend beyond the mere reduction of Aβ aggregation. Its ability to protect against Aβ-mediated oxidative damage [65,72,73,74] and potentially reduce Tau hyperphosphorylation [68,75,76] indicates a comprehensive neuroprotective profile. This suggests that melatonin does not target a single aspect of AD pathology but can mitigate multiple key hallmarks (Aβ, Tau, oxidative stress, and inflammation) that are often interconnected. This extensive efficacy profile suggests that melatonin is a promising multitarget therapeutic agent for AD, with the potential to slow disease progression by targeting multiple critical pathological pathways simultaneously. Its pleiotropic effects could prove to be of significant value in the treatment of complex, multifactorial diseases such as AD.

#### 3.2.3. Enhancement of Amyloid Clearance Pathways

Melatonin is an indispensable molecule in the glymphatic brain-cleaning system, which is responsible for the clearance of amyloid aggregates and metabolic waste from the brain [63,77]. It augments the solubilizing effect of ATP to ensure timely and optimal disaggregation and clearance of pathogenic amyloid aggregates [63,64]. Maintaining protein solubility is a key factor in the effective clearance and efflux of amyloids from the brain, thereby preventing their crystallization [63,64,77]. Glymphatic system dysfunction is directly associated with impaired clearance and subsequent deposition of insoluble amyloid crystals, contributing to the progression of neurodegenerative disease [63,64,77].

Exogenous melatonin not only prevents mitochondrial dysfunction but also elevates ATP production [63,78,79]. High levels of ATP, a biphasic modulator of biomolecular condensates, are crucial for solubilizing and removing amyloid aggregates, particularly during slow-wave sleep [63,64]. Beyond the glymphatic system, melatonin treatment enhances Aβ clearance via the lymphatic system in transgenic mouse models (Tg2576) of amyloidosis [77]. This treatment led to a statistically significant increase in soluble monomeric Aβ40 and a trend toward increased Aβ42 in cervical and axillary lymph nodes, which serve as a surrogate marker of lymphatic clearance [77]. Concurrently, significant decreases in oligomeric Aβ40 were observed in the brain [77]. These findings confirm the active participation of the peripheral lymphatic system in Aβ clearance from the brain and melatonin’s role in augmenting this process. The research reveals that melatonin supports amyloid clearance through a dual, interconnected system: the central glymphatic [63] and the peripheral lymphatic systems [77]. The glymphatic system is critical for the initial solubilization and efflux of amyloid aggregates from the brain parenchyma, a process that is enhanced by melatonin’s role in ATP production and its presence during sleep. Subsequently, the lymphatic system serves as an outflow pathway, removing soluble amyloids from the brain to the peripheral lymph nodes. This finding suggests the presence of a comprehensive multistage clearance mechanism orchestrated, in part, by melatonin. This integrated perspective on amyloid clearance underscores the necessity for effective therapeutic strategies targeting both central brain clearance mechanisms (e.g., glymphatic flow and protein solubilization) and peripheral drainage pathways (e.g., lymphatic function). By influencing both mechanisms, melatonin offers a unique advantage in promoting overall amyloid homeostasis. This finding underscores that systemic factors (e.g., lymphatic health) can significantly affect brain amyloid burden.

#### 3.2.4. Mitigation of Downstream Pathological Events

Melatonin is a potent antioxidant and anti-inflammatory agent that helps mitigate the secondary damage caused by amyloid aggregates [16,80,81]. It reduces abnormal protein nitration, a marker of nitrosative stress [65], and generally reduces inflammation and oxidative stress [68,75,76], which are key drivers of neurodegeneration and β-cell damage. Melatonin may counteract amyloid toxicity through a dual mechanism: by being a radical scavenger and by influencing the interaction between Aβ and lipid membranes [15,82,83]. These studies indicate that melatonin can reduce aggregate formation at the lipid membrane surface and alter the positioning of Aβ aggregates within the lipid bilayer. This finding suggests that melatonin may influence molecular interactions between early-stage Aβ aggregates and the lipid bilayer, a crucial mechanism underlying its protective effects against membrane disruption and subsequent cellular toxicity [15,82,83].

Table 2 summarizes all the information regarding melatonin’s anti-amyloidogenic action.

## 4. Recent Advances and Translational Perspectives

### 4.1. Main Findings from In Vitro and Animal Model Studies

The last five years have brought significant advancements in understanding the complex interplay between IAPP and Aβ. Studies have shown that soluble IAPP significantly accelerates Aβ aggregation, providing a direct molecular link between T2D and AD [47,50,52,54,55]. Concurrently, detailed molecular dynamics simulations have elucidated the precise mechanisms of action of melatonin in inhibiting hIAPP oligomerization, demonstrating its ability to prevent β-sheet and hydrogen-bond formation and to remodel oligomeric conformations [70,71]. The atomic-level resolution provided by recent computational studies represents a major leap forward, moving beyond correlative observations to reveal the specific hydrogen bonding, aromatic stacking, and CH–π interactions that underpin melatonin’s inhibitory effects on IAPP aggregation. This detailed understanding of the preferential binding of melatonin to the hIAPP20–29 amyloidogenic region offers crucial insights for rational drug design [70,71].

Robust in vivo evidence from transgenic mouse models of Alzheimer’s amyloidosis (e.g., Tg2576 mice) has further confirmed the therapeutic potential of melatonin. These studies have shown that melatonin administration partially inhibits the time-dependent elevation of brain Aβ [65,72,73,74] and enhances Aβ lymphatic clearance, leading to increased soluble Aβ in lymph nodes and reduced oligomeric Aβ in the brain [77]. Melatonin has also demonstrated neuroprotective effects in animal models of intracerebral hemorrhage, improving behavioral and pathological outcomes [16]. These animal studies provide compelling evidence for the efficacy of melatonin in mitigating amyloid pathology and the associated neurological deficits in vivo.

### 4.2. Emerging Human Studies and Clinical Trials

Translating research findings from in vitro and animal models directly into clinical settings is challenging. The direct study of human pancreatic β-cells is inherently difficult, often relying on cadaveric pancreatic tissue samples, which makes it challenging to assess real-time abnormalities in β-cell proliferation or regeneration in living individuals.

Nevertheless, there has been renewed interest in the potential association between melatonin and T2D in human physiological studies, spurred by genetic–epidemiological findings. Mutations in melatonin receptor genes have been linked to an increased risk of T2D, and low endogenous melatonin production has been associated with T2D risk [84,85,86,87,88,89]. The present study is an ongoing clinical trial to determine whether the administration of synthetic melatonin induces physiological alterations that may affect the risk of T2D onset. The objective of the present study is to determine whether administration of synthetic melatonin induces physiological changes that affect the risk of developing T2D. One study assessed the acute effects of melatonin on insulin secretion and insulin sensitivity using a Botnia clamp, as well as its potential effects on the incretin response during an oral glucose tolerance test [85,86]. These trials are crucial for understanding the complex influence of melatonin on glucose homeostasis in humans, particularly given the observed inverse relationship between melatonin levels and insulin secretion in animal models [84]. Although these studies focused on the general metabolic effects of melatonin, there is a need for further human research specifically investigating its impact on IAPP aggregation and β-cell preservation in patients with T2D. Further research is warranted to fully explore its anti-amyloidogenic potential in clinical settings.

## 5. Conclusions

IAPP aggregation represents a significant and multifaceted challenge to human health and plays a dual pathological role in both T2D and AD. In T2D, IAPP aggregation, particularly involving toxic, unprocessed proIAPP forms, directly contributes to pancreatic β-cell dysfunction and death through mechanisms including membrane disruption, oxidative stress, ER stress, and inflammation. Crucially, amyloid deposition precedes the onset of hyperglycemia, indicating a causative role exacerbated by metabolic overload. In AD, IAPP contributes to neurodegeneration by impairing the BBB, directly interacting with Aβ to accelerate Aβ aggregation, and promoting neuroinflammation, thereby supporting the “T3D” hypothesis. The common structural features of toxic amyloid aggregates in different diseases suggest their potential as broad-spectrum therapeutic agents.

**Figure 1:**
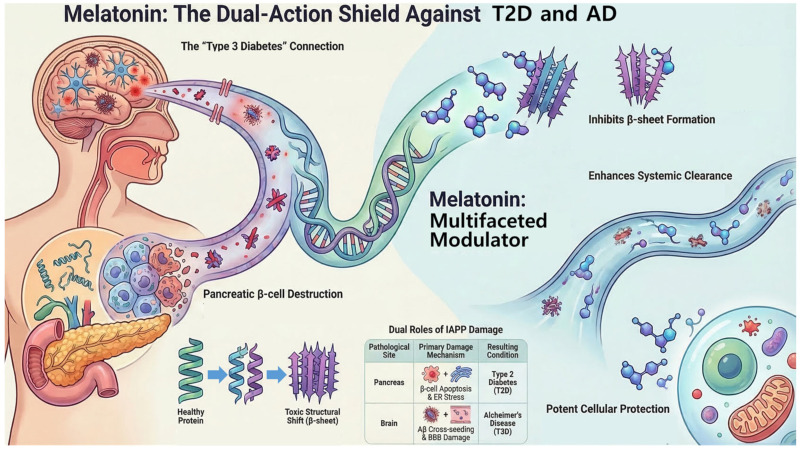
Summary of IAPP aggregation and its melatonin suppression.

Melatonin is a promising, multifaceted modulator of amyloid pathology. Its anti-amyloidogenic effects are diverse, encompassing the direct molecular inhibition of IAPP and Aβ aggregation by preventing β-sheet formation and remodeling oligomeric structures. Furthermore, melatonin significantly enhances amyloid clearance pathways, notably by promoting the glymphatic brain-cleaning system during sleep and by augmenting peripheral lymphatic clearance of Aβ. Melatonin exerts broad neuroprotective effects beyond direct aggregation inhibition by acting as a potent antioxidant and anti-inflammatory agent, thereby mitigating downstream pathological events, including oxidative damage and Tau hyperphosphorylation. However, its complex and sometimes inhibitory influence on pancreatic insulin secretion necessitates careful consideration in clinical applications for T2D (Figure 1).

Future research should prioritize several key areas:

■ Multitarget therapeutic strategies: Given the interconnected pathological network underlying IAPP toxicity, future interventions should explore multitarget approaches that combine melatonin with other agents. This may involve drugs that enhance proteostasis, reduce inflammation, or disaggregate mature fibrils, thereby creating synergistic therapeutic effects.
■ Personalized medical approaches: The nuanced role of melatonin in glucose homeostasis, which is influenced by genetic variations in melatonin receptors and endogenous production, suggests a need for personalized dosing strategies. Future studies should investigate how genetic profiling or real-time monitoring of endogenous melatonin levels can guide therapeutic interventions, particularly in individuals with T2D.
■ Circadian rhythm management: Recognizing the role of melatonin as a proteostatic orchestrator of circadian rhythms and sleep, future therapeutic strategies for amyloidopathies should integrate interventions aimed at restoring healthy sleep patterns. This may involve chronotherapeutic approaches and direct pharmacological interventions.
■ Targeting early-stage aggregation: The demonstrated efficacy of melatonin in preventing early-stage oligomerization highlights the importance of early intervention. Research should focus on identifying biomarkers of early amyloid accumulation to develop preventive strategies before extensive fibril deposition occurs.
■ Robust human clinical trials: Although promising animal data exist, more rigorous and comprehensive human clinical trials are required. These trials should specifically investigate melatonin’s effects on IAPP aggregation and β-cell function in patients with T2D, as well as its neuroprotective effects in AD, with careful monitoring of both amyloid biomarkers and metabolic parameters.

While some conclusions may be controversial, advancing this field of research can lead to a deeper understanding of amyloid pathogenesis and the identification of melatonin’s therapeutic potential, laying the foundation for new and effective strategies to combat debilitating diseases.

## Figures and Tables

**Table 1:** Molecular and cellular mechanisms of IAPP-induced pathogenesis.

Mechanism Classification	Specific Mechanism	Primary Impact/Consequence	References
Protein misfolding & aggregation	Formation of toxic oligomers and protofibrils; Accumulation of unprocessed proIAPP forms (seeding); Fibril deposition.	β-cell death/dysfunction; Impaired insulin secretion; Neurotoxicity/cognitive decline; Diabetes-associated dementia.	[5]
Cellular stress & dysfunction	Membrane disruption and ion channel formation; Generation of ROS and oxidative stress; ER stress and Unfolded Protein Response; Proteasome dysfunction; Defects in autophagy; β-cell apoptosis; Synaptic dysfunction (in the brain).		[32,35]
Inflammation & immune response	Activation of the inflammasome; Promotion of an inflammatory environment; Glial cell activation (in the brain).		[32,35]
Inter-protein interactions & systemic effects	Impairment of BBB; Direct interaction and cross-seeding with Aβ; Modulation of neuronal receptors (e.g., AMY3); Exacerbation by metabolic stressors (glucotoxicity, lipotoxicity, and insulin resistance).		[11,12,41,42]

**Table 2:** Melatonin’s multifaceted mechanisms of action in amyloid inhibition and clearance.

Mechanism Classification	Specific Mechanism	Target Amyloid/System	References
Direct inhibition of aggregation	Prevents β-sheet and backbone hydrogen bond formation (hIAPP); Remodels hIAPP oligomers (less compact, more disordered); Preferentially binds hIAPP20–29 via H-bonding, aromatic stacking, CH–π interactions; Inhibits Aβ fibril formation; Alters Aβ interaction with lipid membranes.	hIAPP; Aβ; Lipid membranes.	[15,70,71,82,83]
Modulation of downstream pathology	Potent antioxidant (radical scavenger); Reduces oxidative damage (e.g., Aβ-mediated, protein nitration); Potent anti-inflammatory agent; Reduces TAU protein hyperphosphorylation.	Neuronal cells; Pancreatic β-cells; Aβ; Tau.	[15,68,75,76]
Enhancement of clearance pathways	Indispensable molecule in glymphatic brain-cleaning system (augments solubilization/efflux); Prevents mitochondrial dysfunction and elevates ATP production (aids solubilization); Enhances lymphatic Aβ clearance (increases soluble Aβ in lymph nodes, reduces brain oligomers).	Brain (Glymphatic system); Peripheral Lymphatic system; Mitochondria.	[63,68,79]

## Data Availability

The original contributions presented in this study are included in the article. Further inquiries can be directed to the corresponding authors.

## References

[1] Ross C.A., Poirier M.A. (2004). Protein aggregation and neurodegenerative disease. Nat. Med..

[2] Westermark G.T., Fändrich M., Westermark P. (2015). AA amyloidosis: Pathogenesis and targeted therapy. Annu. Rev. Pathol..

[3] Biancalana M., Makabe K., Koide A., Koide S. (2009). Molecular mechanism of thioflavin-T binding to the surface of beta-rich peptide self-assemblies. J. Mol. Biol..

[4] Duboisset J., Ferrand P., He W., Wang X., Rigneault H., Brasselet S. (2013). Thioflavine-T and Congo Red reveal the polymorphism of insulin amyloid fibrils when probed by polarization-resolved fluorescence microscopy. J. Phys. Chem. B.

[5] Hull R.L., Westermark G.T., Westermark P., Kahn S.E. (2004). Islet amyloid: A critical entity in the pathogenesis of type 2 diabetes. J. Clin. Endocrinol. Metab..

[6] Ghosh P., Kumar A., Datta B., Rangachari V. (2010). Dynamics of protofibril elongation and association involved in Aβ42 peptide aggregation in Alzheimer’s disease. BMC Bioinform..

[7] Foster J.S., Balachandran M., Hancock T.J., Martin E.B., Macy S., Wooliver C., Richey T., Stuckey A., Williams A.D., Jackson J.W. (2023). Development and characterization of a prototypic pan-amyloid clearing agent—A novel murine peptide-immunoglobulin fusion. Front. Immunol..

[8] Andrikopoulos N., Tang H., Wang Y., Liang X., Li Y., Davis T.P., Ke P.C. (2024). Exploring Peptido-Nanocomposites in the Context of Amyloid Diseases. Angew. Chem. Int. Ed. Engl..

[9] Uversky V.N. (2010). Mysterious oligomerization of the amyloidogenic proteins. FEBS J..

[10] Fortin J.S., Benoit-Biancamano M.O. (2016). Inhibition of islet amyloid polypeptide aggregation and associated cytotoxicity by nonsteroidal anti-inflammatory drugs. Can. J. Physiol. Pharmacol..

[11] Raimundo A.F., Ferreira S., Martins I.C., Menezes R. (2020). Islet Amyloid Polypeptide: A Partner in Crime with Aβ in the Pathology of Alzheimer’s Disease. Front. Mol. Neurosci..

[12] Westermark P., Andersson A., Westermark G.T. (2011). Islet amyloid polypeptide, islet amyloid, and diabetes mellitus. Physiol. Rev..

[13] Jackson K., Barisone G.A., Diaz E., Jin L.W., DeCarli C., Despa F. (2013). Amylin deposition in the brain: A second amyloid in Alzheimer disease?. Ann. Neurol..

[14] Ly H., Verma N., Wu F., Liu M., Saatman K.E., Nelson P.T., Slevin J.T., Goldstein L.B., Biessels G.J., Despa F. (2017). Brain microvascular injury and white matter disease provoked by diabetes-associated hyperamylinemia. Ann. Neurol..

[15] Mei N., Liang J., McRae D.M., Leonenko Z. (2024). Localized surface plasmon resonance and atomic force microscopy study of model lipid membranes and their interactions with amyloid and melatonin. Nanotechnology.

[16] Zeng L., Zhu Y., Hu X., Qin H., Tang J., Hu Z., Chen C. (2021). Efficacy of melatonin in animal models of intracerebral hemorrhage: A systematic review and meta-analysis. Aging.

[17] Pittner R.A., Albrandt K., Beaumont K., Gaeta L.S., Koda J.E., Moore C.X., Rittenhouse J., Rink T.J. (1994). Molecular physiology of amylin. J. Cell. Biochem..

[18] Koopmans S.J., van Mansfeld A.D., Jansz H.S., Krans H.M., Radder J.K., Frölich M., de Boer S.F., Kreutter D.K., Andrews G.C., Maassen J.A. (1991). Amylin-induced in vivo insulin resistance in conscious rats: The liver is more sensitive to amylin than peripheral tissues. Diabetologia.

[19] Rushing P.A., Hagan M.M., Seeley R.J., Lutz T.A., D’Alessio D.A., Air E.L., Woods S.C. (2001). Inhibition of central amylin signaling increases food intake and body adiposity in rats. Endocrinology.

[20] Araújo A.R., Reis R.L., Pires R.A. (2020). Natural Polyphenols as Modulators of the Fibrillization of Islet Amyloid Polypeptide. Adv. Exp. Med. Biol..

[21] Muskiet M.H.A., Nardone M., Rensen P.C.N., Cherney D.Z.I., Cooper M.E. (2026). Amylin and the renin-angiotensin system: Risk or opportunity in amylin-based therapy?. Lancet.

[22] El Assar M., Angulo J., Santos-Ruiz M., Moreno P., Novials A., Villanueva-Peñacarrillo M.L., Rodríguez-Mañas L. (2015). Differential effect of amylin on endothelial-dependent vasodilation in mesenteric arteries from control and insulin resistant rats. PLoS ONE.

[23] Bortoletto A.S., Graham W.V., Trout G., Bonito-Oliva A., Kazmi M.A., Gong J., Weyburne E., Houser B.L., Sakmar T.P., Parchem R.J. (2022). Human Islet Amyloid Polypeptide (hIAPP) Protofibril-Specific Antibodies for Detection and Treatment of Type 2 Diabetes. Adv. Sci..

[24] Pang B., Zhuang X., Bian X., Liu S., Liu Z., Song F. (2020). Studies on the cross-interaction between hIAPP and Aβ25-35 and the aggregation process in binary mixture by electrospray ionization-ion mobility-mass spectrometry. J. Mass Spectrom..

[25] Rehn F., Kraemer-Schulien V., Bujnicki T., Bannach O., Tschoepe D., Stratmann B., Willbold D. (2024). IAPP—Oligomerisation levels in plasma of people with type 2 diabetes. Sci. Rep..

[26] Gurlo T., Liu R., Wang Z., Hoang J., Ryazantsev S., Daval M., Butler A.E., Yang X., Blencowe M., Butler P.C. (2024). Dysregulation of cholesterol homeostasis is an early signal of β-cell proteotoxicity characteristic of type 2 diabetes. Physiol. Genom..

[27] Mukherjee A., Morales-Scheihing D., Salvadores N., Moreno-Gonzalez I., Gonzalez C., Taylor-Presse K., Mendez N., Shahnawaz M., Gaber A.O., Sabek O.M. (2017). Induction of IAPP amyloid deposition and associated diabetic abnormalities by a prion-like mechanism. J. Exp. Med..

[28] Pithadia A., Brender J.R., Fierke C.A., Ramamoorthy A. (2016). Inhibition of IAPP Aggregation and Toxicity by Natural Products and Derivatives. J. Diabetes Res..

[29] Dubey R., Kulkarni S.H., Dantu S.C., Panigrahi R., Sardesai D.M., Malik N., Acharya J.D., Chugh J., Sharma S., Kumar A. (2020). Myricetin protects pancreatic β-cells from human islet amyloid polypeptide (hIAPP) induced cytotoxicity and restores islet function. Biol. Chem..

[30] Roy R., Paul S. (2020). Theoretical Investigation of the Inhibitory Mechanism of Norepinephrine on hIAPP Amyloid Aggregation and the Destabilization of Protofibrils. J. Phys. Chem. B.

[31] Moya-Gudiño V., Altamirano-Bustamante N.F., Revilla-Monsalve C., Altamirano-Bustamante M.M. (2025). Decoding the Contribution of IAPP Amyloid Aggregation to Beta Cell Dysfunction: A Systematic Review and Epistemic Meta-Analysis of Type 1 Diabetes. Int. J. Mol. Sci..

[32] Brender J.R., Salamekh S., Ramamoorthy A. (2012). Membrane disruption and early events in the aggregation of the diabetes related peptide IAPP from a molecular perspective. Acc. Chem. Res..

[33] Marmentini C., Branco R.C.S., Boschero A.C., Kurauti M.A. (2022). Islet amyloid toxicity: From genesis to counteracting mechanisms. J. Cell. Physiol..

[34] Mirzabekov T.A., Lin M.C., Kagan B.L. (1996). Pore formation by the cytotoxic islet amyloid peptide amylin. J. Biol. Chem..

[35] Akter R., Cao P., Noor H., Ridgway Z., Tu L.H., Wang H., Wong A.G., Zhang X., Abedini A., Schmidt A.M. (2016). Islet Amyloid Polypeptide: Structure, Function, and Pathophysiology. J. Diabetes Res..

[36] Costes S., Gurlo T., Rivera J.F., Butler P.C. (2014). UCHL1 deficiency exacerbates human islet amyloid polypeptide toxicity in β-cells: Evidence of interplay between the ubiquitin/proteasome system and autophagy. Autophagy.

[37] Shigihara N., Fukunaka A., Hara A., Komiya K., Honda A., Uchida T., Abe H., Toyofuku Y., Tamaki M., Ogihara T. (2014). Human IAPP-induced pancreatic β cell toxicity and its regulation by autophagy. J. Clin. Investig..

[38] Caruso G., Distefano D.A., Parlascino P., Fresta C.G., Lazzarino G., Lunte S.M., Nicoletti V.G. (2017). Receptor-mediated toxicity of human amylin fragment aggregated by short- and long-term incubations with copper ions. Mol. Cell. Biochem..

[39] Matveyenko A.V., Butler P.C. (2006). Islet amyloid polypeptide (IAPP) transgenic rodents as models for type 2 diabetes. ILAR J..

[40] Steiner D.F., James D.E. (1992). Cellular and molecular biology of the beta cell. Diabetologia.

[41] Chen Y.C., Taylor A.J., Verchere C.B. (2018). Islet prohormone processing in health and disease. Diabetes Obes. Metab..

[42] Ramzy A., Kieffer T.J. (2022). Altered islet prohormone processing: A cause or consequence of diabetes?. Physiol. Rev..

[43] Abedini A., Tracz S.M., Cho J.H., Raleigh D.P. (2006). Characterization of the heparin binding site in the N-terminus of human pro-islet amyloid polypeptide: Implications for amyloid formation. Biochemistry.

[44] Khemtemourian L., Antoniciello F., Sahoo B.R., Decossas M., Lecomte S., Ramamoorthy A. (2021). Investigation of the effects of two major secretory granules components, insulin and zinc, on human-IAPP amyloid aggregation and membrane damage. Chem. Phys. Lipids.

[45] Meng F., Raleigh D.P. (2011). Inhibition of glycosaminoglycan-mediated amyloid formation by islet amyloid polypeptide and proIAPP processing intermediates. J. Mol. Biol..

[46] Jaikaran E.T., Clark A. (2001). Islet amyloid and type 2 diabetes: From molecular misfolding to islet pathophysiology. Biochim. Biophys. Acta.

[47] Deng J., Liu B., Tao Q., Luo Y., Zhu Y., Huang X., Yue F. (2024). The Co-oligomers of Aβ42 and Human Islet Amyloid Polypeptide Exacerbate Neurotoxicity and Alzheimer-like Pathology at Cellular Level. Neuroscience.

[48] Moreno-Gonzalez I., Edwards G., Salvadores N., Shahnawaz M., Diaz-Espinoza R., Soto C. (2017). Molecular interaction between type 2 diabetes and Alzheimer’s disease through cross-seeding of protein misfolding. Mol. Psychiatry.

[49] Nuñez-Diaz C., Andersson E., Schultz N., Pocevičiūtė D., Hansson O., Netherland Brain Bank, Nilsson K.P.R., Wennström M. (2024). The fluorescent ligand bTVBT2 reveals increased p-tau uptake by retinal microglia in Alzheimer’s disease patients and AppNL-F/NL-F mice. Alzheimers Res. Ther..

[50] Stanciu G.D., Bild V., Ababei D.C., Rusu R.N., Cobzaru A., Paduraru L., Bulea D. (2020). Link Between Diabetes and Alzheimer’s Disease due to the Shared Amyloid Aggregation and Deposition Involving both Neurodegenerative Changes and Neurovascular Damages. J. Clin. Med..

[51] Michailidis M., Moraitou D., Tata D.A., Kalinderi K., Papamitsou T., Papaliagkas V. (2022). Alzheimer’s Disease as Type 3 Diabetes: Common Pathophysiological Mechanisms between Alzheimer’s Disease and Type 2 Diabetes. Int. J. Mol. Sci..

[52] Nomoto D., Tsunoda T., Shigemori H. (2021). Effects of clovamide and its related compounds on the aggregations of amyloid polypeptides. J. Nat. Med..

[53] Ferreira S., Raimundo A.F., Menezes R., Martins I.C. (2021). Islet amyloid polypeptide & amyloid beta peptide roles in Alzheimer’s disease: Two triggers, one disease. Neural Regen. Res..

[54] Siddiqui F., Mishra P., Khanam S., Ranjan S., Alam P., Albalawi T., Khan S., Mir S.S. (2025). Nano-Chaperones: Bridging Therapeutics for Amyloid Aggregation in Alzheimer’s Disease and Type-2 Diabetes Mellitus. Eur. J. Neurosci..

[55] Ge X., Yang Y., Sun Y., Cao W., Ding F. (2018). Islet Amyloid Polypeptide Promotes Amyloid-Beta Aggregation by Binding-Induced Helix-Unfolding of the Amyloidogenic Core. ACS Chem. Neurosci..

[56] Aftabizadeh M., Tatarek-Nossol M., Andreetto E., El Bounkari O., Kipp M., Beyer C., Latz E., Bernhagen J., Kapurniotu A. (2019). Blocking Inflammasome Activation Caused by β-Amyloid Peptide (Aβ) and Islet Amyloid Polypeptide (IAPP) through an IAPP Mimic. ACS Chem. Neurosci..

[57] Casas S., Novials A., Reimann F., Gomis R., Gribble F.M. (2008). Calcium elevation in mouse pancreatic beta cells evoked by extracellular human islet amyloid polypeptide involves activation of the mechanosensitive ion channel TRPV4. Diabetologia.

[58] Kimura R., MacTavish D., Yang J., Westaway D., Jhamandas J.H. (2017). Pramlintide Antagonizes Beta Amyloid (Aβ)- and Human Amylin-Induced Depression of Hippocampal Long-Term Potentiation. Mol. Neurobiol..

[59] Brown G.M. (1994). Light, melatonin and the sleep-wake cycle. J. Psychiatry Neurosci..

[60] Claustrat B., Leston J. (2015). Melatonin: Physiological effects in humans. Neurochirurgie.

[61] Gobetti R.A.P., Bueno C., Soster L.M.S.F.A., Monazzi A.C.C.B.L., do Amaral F.G., Capellano A.M., da Silva N.S., Cipolla-Neto J. (2025). Sleep and Rhythmic Profile After Pineal Gland Removal in Humans. J. Sleep Res..

[62] Pevet P., Challet E., Felder-Schmittbuhl M.P. (2021). Melatonin and the circadian system: Keys for health with a focus on sleep. Handb. Clin. Neurol..

[63] Loh D., Reiter R.J. (2025). Melatonin regulation of phase separation in Neuro-PASC: Out-maneuvering Janus-faced amyloids. Explor. Neurosci..

[64] Lucke-Wold B.P., Smith K.E., Nguyen L., Turner R.C., Logsdon A.F., Jackson G.J., Huber J.D., Rosen C.L., Miller D.B. (2015). Sleep disruption and the sequelae associated with traumatic brain injury. Neurosci. Biobehav. Rev..

[65] Matsubara E., Bryant-Thomas T., Pacheco Quinto J., Henry T.L., Poeggeler B., Herbert D., Cruz-Sanchez F., Chyan Y.J., Smith M.A., Perry G. (2003). Melatonin increases survival and inhibits oxidative and amyloid pathology in a transgenic model of Alzheimer’s disease. J. Neurochem..

[66] Pappolla M.A., Chyan Y.J., Poeggeler B., Bozner P., Ghiso J., LeDoux S.P., Wilson G.L. (1999). Alzheimer beta protein mediated oxidative damage of mitochondrial DNA: Prevention by melatonin. J. Pineal Res..

[67] Pappolla M.A., Simovich M.J., Bryant-Thomas T., Chyan Y.J., Poeggeler B., Dubocovich M., Bick R., Perry G., Cruz-Sanchez F., Smith M.A. (2002). The neuroprotective activities of melatonin against the Alzheimer beta-protein are not mediated by melatonin membrane receptors. J. Pineal Res..

[68] Andrade M.K., Souza L.C., Azevedo E.M., Bail E.L., Zanata S.M., Andreatini R., Vital M.A.B.F. (2023). Melatonin reduces β-amyloid accumulation and improves short-term memory in streptozotocin-induced sporadic Alzheimer’s disease model. IBRO Neurosci. Rep..

[69] Qi R., Wei G., Ma B., Nussinov R. (2018). Replica Exchange Molecular Dynamics: A Practical Application Protocol with Solutions to Common Problems and a Peptide Aggregation and Self-Assembly Example. Methods Mol. Biol..

[70] Wang G., Zhu X., Song X., Zhang Q., Qian Z. (2022). Melatonin Inhibits hIAPP Oligomerization by Preventing beta-Sheet and Hydrogen Bond Formation of the Amyloidogenic Region Revealed by Replica-Exchange Molecular Dynamics Simulation. Int. J. Mol. Sci..

[71] Porat Y., Mazor Y., Efrat S., Gazit E. (2004). Inhibition of islet amyloid polypeptide fibril formation: A potential role for heteroaromatic interactions. Biochemistry.

[72] Goleij P., Khazeei Tabari M.A., Poudineh M., Sanaye P.M., Khan H., Kumar A.P., Larsen D.S., Daglia M. (2025). Therapeutic potential of melatonin-induced mitophagy in the pathogenesis of Alzheimer’s disease. Inflammopharmacology.

[73] Salgado K.D.C.B., Nascimento R.G.F., Albuquerque A.L.S., Oliveira L.A.M., Pinto Coelho Nogueira K.O. (2025). Melatonin protects mouse hippocampal neurons from neurotoxicity induced by amyloid β-peptide25-35. Brain Res..

[74] Sirimaharaj N., Thiankhaw K., Chattipakorn N., Chattipakorn S.C. (2025). Unveiling the Protective Roles of Melatonin on Glial Cells in the Battle Against Alzheimer’s Disease-Insights from In Vivo and In Vitro Studies. Mol. Neurobiol..

[75] Chen D., Lan G., Li R., Mei Y., Shui X., Gu X., Wang L., Zhang T., Gan C.L., Xia Y. (2022). Melatonin ameliorates tau-related pathology via the miR-504-3p and CDK5 axis in Alzheimer’s disease. Transl. Neurodegener..

[76] Panmanee J., Phanchana M., Pearngam P., Petchyam N., Promthep K., Wisomka P., Kutpruek S., Pannengpetch S., Prasertporn T., Mukda S. (2025). A Proteomics Profiling Reveals the Neuroprotective Effects of Melatonin on Exogenous β-amyloid-42 Induced Mitochondrial Impairment, Intracellular β-amyloid Accumulation and Tau Hyperphosphorylation in Human SH-SY5Y Cells. Cell Biol. Int..

[77] Pappolla M.A., Matsubara E., Vidal R., Pacheco-Quinto J., Poeggeler B., Zagorski M., Sambamurti K. (2018). Melatonin Treatment Enhances Aβ Lymphatic Clearance in a Transgenic Mouse Model of Amyloidosis. Curr. Alzheimer Res..

[78] Asghari M.H., Abdollahi M., de Oliveira M.R., Nabavi S.M. (2017). A review of the protective role of melatonin during phosphine-induced cardiotoxicity: Focus on mitochondrial dysfunction, oxidative stress and apoptosis. J. Pharm. Pharmacol..

[79] Jauhari A., Monek A.C., Suofu Y., Amygdalos O.R., Singh T., Baranov S.V., Carlisle D.L., Friedlander R.M. (2025). Melatonin Deficits Result in Pathologic Metabolic Reprogramming in Differentiated Neurons. J. Pineal Res..

[80] Hoppe J.B., Frozza R.L., Horn A.P., Comiran R.A., Bernardi A., Campos M.M., Battastini A.M.O., Salbego C. (2010). Amyloid-beta neurotoxicity in organotypic culture is attenuated by melatonin: Involvement of GSK-3beta, tau and neuroinflammation. J. Pineal Res..

[81] Jürgenson M., Zharkovskaja T., Noortoots A., Morozova M., Beniashvili A., Zapolski M., Zharkovsky A. (2019). Effects of the drug combination memantine and melatonin on impaired memory and brain neuronal deficits in an amyloid-predominant mouse model of Alzheimer’s disease. J. Pharm. Pharmacol..

[82] Dies H., Toppozini L., Rheinstädter M.C. (2014). The interaction between amyloid-β peptides and anionic lipid membranes containing cholesterol and melatonin. PLoS ONE.

[83] Rosales-Corral S.A., Lopez-Armas G., Cruz-Ramos J., Melnikov V.G., Tan D.X., Manchester L.C., Munoz R., Reiter R.J. (2012). Alterations in Lipid Levels of Mitochondrial Membranes Induced by Amyloid-β: A Protective Role of Melatonin. Int. J. Alzheimers Dis..

[84] Peschke E., Bähr I., Mühlbauer E. (2013). Melatonin and pancreatic islets: Interrelationships between melatonin, insulin and glucagon. Int. J. Mol. Sci..

[85] Lauritzen E.S., Stoy J., Baech-Laursen C., Grarup N., Jessen N., Hansen T., Moller N., Hartmann B., Holst J.J., Kampmann U. (2021). The Effect of Melatonin on Incretin Hormones: Results from Experimental and Randomized Clinical Studies. J. Clin. Endocrinol. Metab..

[86] Kampmann U., Lauritzen E.S., Grarup N., Jessen N., Hansen T., Moller N., Stoy J. (2021). Acute metabolic effects of melatonin-A randomized crossover study in healthy young men. J. Pineal Res..

[87] Huang Y., Dou X., He M., Su Y., Lin H., Yang Y. (2025). The G-allele of rs10830963 in MTNR1B Exerts Stage-Specific Effects Across the Trajectory of Type 2 Diabetes: A Multi-State Analysis. Int. J. Mol. Sci..

[88] Lane J.M., Chang A.M., Bjonnes A.C., Aeschbach D., Anderson C., Cade B.E., Cain S.W., Czeisler C.A., Gharib S.A., Gooley J.J. (2016). Impact of Common Diabetes Risk Variant in MTNR1B on Sleep, Circadian, and Melatonin Physiology. Diabetes.

[89] Lyssenko V., Nagorny C.L., Erdos M.R., Wierup N., Jonsson A., Spégel P., Bugliani M., Saxena R., Fex M., Pulizzi N. (2009). Common variant in MTNR1B associated with increased risk of type 2 diabetes and impaired early insulin secretion. Nat. Genet..

